# The Emerging Role of Cell Adhesion Molecules on Benign Prostatic Hyperplasia

**DOI:** 10.3390/ijms24032870

**Published:** 2023-02-02

**Authors:** Jiang Liu, Junchao Zhang, Xun Fu, Shu Yang, Yan Li, Jianmin Liu, Michael E. DiSanto, Ping Chen, Xinhua Zhang

**Affiliations:** 1 Department of Urology, Zhongnan Hospital of Wuhan University, Wuhan 430071, China; 2 Department of Surgery and Biomedical Sciences, Cooper Medical School of Rowan University, Camden, NJ 08103, USA

**Keywords:** cell adhesion molecules, benign prostatic hyperplasia, cadherins, integrins

## Abstract

Benign prostatic hyperplasia (BPH) is a common disease in elderly men. It is characterized by prostatic enlargement and urethral compression and often causes lower urinary tract symptoms (LUTs) such as urinary frequency, urgency, and nocturia. Existing studies have shown that the pathological process of prostate hyperplasia is mainly related to the imbalance of cell proliferation and apoptosis, inflammation, epithelial–mesenchymal transition (EMT), and growth factors. However, the exact molecular mechanisms remain incompletely elucidated. Cell adhesion molecules (CAMs) are a group of cell surface proteins that mediate cell–cell adhesion and cell migration. Modulating adhesion molecule expression can regulate cell proliferation, apoptosis, EMT, and fibrotic processes, engaged in the development of prostatic hyperplasia. In this review, we went over the important roles and molecular mechanisms of cell adhesion molecules (mainly integrins and cadherins) in both physiological and pathological processes. We also analyzed the mechanisms of CAMs in prostate hyperplasia and explored the potential value of targeting CAMs as a therapeutic strategy for BPH.

## 1. Introduction

Benign prostatic hyperplasia (BPH) is a common disease worldwide afflicting elderly males. It often presents lower urinary tract symptoms (LUTs), such as urinary frequency, urgency, nocturia, and incontinence, which greatly reduces the health level and life quality [1]. The incidence of BPH increases with age, reaching 88% by the age of 80 years old [2]. Prostatic hyperplasia is characterized by increased numbers of epithelial and stromal cells in the periurethral region of the prostate [3]. It is believed that this process may be associated with androgen/estrogen dysregulation, stromal epithelial interactions, growth factors, and inflammation [4,5]. However, the exact molecular etiology of the hyperplastic process is uncertain.

Cell adhesion molecules (CAMs) are a large family of cell surface proteins, which are generally classified into four small families: selectins, integrins, cadherins, and members of the Ig superfamily of CAMs (IgCAMs) [6,7,8,9]. In addition to these classical CAMs, non-classical CAMs, such as vascular adhesion protein 1 (VAP-1) [10], the mucosal addressing protein cell adhesion molecule 1 (MAdCAM-1) [11], and stabilizers [12], also have an effect on cell adhesion. CAMs enable cells to interact with other cells and/or with ECM, influencing tissue remodeling, fibrosis, inflammation, and cell survival [13,14,15,16]. In our current review, we concentrated on the roles of cell adhesion molecules (limited to integrins and cadherins) in BPH.

## 2. Overview of BPH

Pathophysiologically, BPH is a hyperplastic but not a hypertrophic process with a net increase in cell number rather than in cell size [17]. BPH is characterized by stromal and epithelial hyperplasia and subsequent nodule formation. Prostate intraepithelial neoplasia (PIN) and PCa mainly arise in the prostate gland from epithelial cells, localized in the peripheral zone [18,19]. Additionally, PIN is generally considered as a putative prostatic cancer precursor [19]. Unlike PCa, McNeal demonstrated that BPH first occurs in the periurethral transition zone of the prostate [20]. The volume and cell number of the prostate gland depend on the balance between cell proliferation and cell death. Organs can expand not only by increased cell proliferation, but also by reduced cell apoptosis [21]. Androgens, growth factors, and cytokines were supposed to contribute to the imbalance of the proliferation/apoptosis ratio of prostate epithelial and stromal cells [22]. In the rat prostate, active cell death naturally occurs in the proximal part of the prostatic ductal system in the presence of normal plasma testosterone concentrations [23], whereas there is an increase in apoptosis of the luminal epithelial population as well as distal regions of the prostatic ductal system after castration, demonstrating the modulatory effect of androgens on cell apoptosis in different parts of the prostate gland. In addition, it was reported that knockdown of androgen receptors (AR) in mice with smooth stromal muscle can reduce the proliferative activity of prostate epithelial cells and the volume of the prostate anterior lobe [24,25]. Indeed, androgens are permissive but insufficient for BPH. Specifically, androgens’ amplification in castrated animals induced prostatic regrowth and enlarged the prostate gland. However, supplementation with androgens does not appear to increase the incident risk of BPH, indicating that androgens may not influence prostate growth [26,27]. Unlike androgens, prostatic overproduction of epithelial AR in mice caused increased prostate proliferation, demonstrating that amplification and hyperactivation of AR can promote the development of BPH [28,29]. Additionally, Evans et al. [30] detected AR mutations in prostate hyperplasia samples, suggesting that AR mutations may also contribute to the onset and progression of BPH.

Moreover, it appears that growth-stimulating factors, such as the basic fibroblast growth factor (bFGF) [31], insulin-like growth factor (IGF) [32], epidermal growth factor (EGF) [33], vascular endothelial growth factor (VEGF) [34], nerve growth factor (NGF) [35], and platelet-derived growth factor (PDGF) [36] also maintain and promote the proliferation of prostate cells. The bFGF, synthesized in fibroblasts of the prostatic stroma, showed a 2-fold increase in the hyperplastic prostate. It could stimulate cell differentiation and proliferation via binding to bFGF receptors with tyrosine kinase activity [31,37]. Consistently, transgenic mice that overexpressed the protein int-2 (a 27-kDa polypeptide, functionally similar to bFGF) developed marked prostatic epithelial hyperplasia in vivo [38]. The growth-promoting effect of IGFs in the prostate was first demonstrated in vitro by Cohen et al., elucidating that IGFs could enhance the growth of prostatic epithelial cells [32]. Moreover, an in vivo model showed that exogenous IGF increased rat prostate size [39]. The production and secretion of EGF were augmented by the presence of circulating androgens, which were important for the maintenance of structural and functional integrity in benign prostatic epithelium [33]. In addition, the prostate size in dogs was positively correlated with VEGF levels in serum, illustrating the promoting effect of VEGF on prostate enlargement [34]. Regarding NGF and PDGF, studies have shown their involvement in the development of prostatic hyperplasia, and revealed that NGF and PDGF production in the prostate is one of the possible etiologies of BPH [35,36]. Additionally, evidence from recent studies suggested that the bone morphogenetic protein 5 (BMP5) [40] and the smoothened (SMO) [41] protein affect cell proliferation and apoptosis by regulating the cell cycle. After inhibiting the expression of SMO in vivo and in vitro, we observed a cell cycle arrest at the G0/G1 phase with a significant increase in apoptosis, accompanied by a decrease of tissue fibrosis markers, such as collagen and α-SMA [41]. Therefore, the interaction among androgens, growth factors, and other molecules (e.g., BMP5, SMO) could modulate the proliferation and cell death of the prostate. The enlarged prostate increases urethral resistance, followed by compensatory changes in bladder and detrusor function, which in turn triggers LUTs. In addition, the presence of a prostatic capsule presumably transmits the “pressure” of tissue expansion to the urethra and leads to increased urethral resistance [2]. The appearance of LUTs usually means that the BPH enters the clinical stage, which impairs quality of life and therefore requires corresponding treatment.

Furthermore, recent studies have showed that EMT plays an important role in BPH, enriching the etiology of prostatic hyperplasia. EMT is a cellular process by which cells lose their epithelial characteristics and acquire a mesenchymal phenotype [42]. EMT has long been considered to be associated with the metastasis and invasion of various tumors, such as prostate cancer [43], pancreatic cancer [44], and breast cancer [45]. However, recent studies have shown that the EMT process is also present in some benign diseases such as BPH [4]. Alonso detected 16 BPH samples and found that: (I) areas where prostatic epithelial cells did not express E-cadherin had lost polarization and became spindle-shaped (nuclei of these cells are strongly positive for pSmad 3 and Snail); and (II) areas where the walls of the blood vessels were extremely thick lost the endothelial layer, which was considered characteristic of EMT [4]. In addition, vimentin expression was significantly increased in the epithelial tissues of BPH, implying EMT was involved in the development of BPH [46]. In recent years, our laboratory observed the changes of N-cadherin, E-cadherin, and vimentin triggered by RNA interference on BMP5, SMO, and interleukin 21 receptor (IL-21R), accompanied by the proliferation/apoptosis imbalance, fibrosis alterations and reactive oxygen species changes in mesenchymal and epithelial cells [22,40,47], which provided strong evidence for the involvement of EMT in the development of BPH. 

Additionally, inflammation was attributed to the development of BPH. The normal prostate is an immunocompetent organ composed of a limited number of inflammatory cells [48]. Chronic inflammation in the prostate may be related to hormonal changes, infections (bacterial or viral), dietary or environmental factors, autoimmune reactions, urinary reflux within the collecting ducts of the prostate, and systemic inflammation associated with metabolic syndrome [49]. When exposed to external stimuli, T lymphocytes, macrophages, and B lymphocytes caused damage to epithelial and stromal cells with an increased release of cytokine and growth factors, which can promote the abnormal remodeling process characterized by fibromuscular growth [49,50].

In addition, fibrosis is an important factor in the aggravation of BPH symptoms [51]. The prostatic hyperplasia gland possessed increased stiffness and thickened collagen in the periurethral transition zone of the prostate, indicating the involvement of fibrosis in the onset and progression of BPH [51,52]. On a cellular level, prostate stromal fibroblasts can be induced to express fibrosis-associated collagen I and III and α-SMA, and to undergo functional myofibroblast phenoconversion in response to exposure to the profibrotic protein TGF-β1 or the CXCLs (CXC-type chemokines secreted by the aging prostate tissue microenvironment) [53].

## 3. Cell Adhesion Molecular Family 

CAMs are mostly transmembrane receptor proteins. They are widely expressed in the normal epithelium, endothelium, and immune cells, and can induce cell-to-cell and cell-to-ECM adhesion. They are composed of three domains: an intracellular domain, a transmembrane domain, and an extracellular domain. The intracellular domain interacts with the cytoskeleton to transduce biological signals, whereas the extracellular domain binds to other CAMs or ECM. In the presence of cell adhesion molecules, extracellular signals integrate with cell-intrinsic signals to influence intracellular responses, cytoskeletal organization, intracellular signaling, and gene expression [54]. In this review, we focus on integrins and cadherins with special attention on their roles in the development of prostatic hyperplasia.

### 3.1. Integrins

#### 3.1.1. The Structure of Integrins

**Figure 1:**
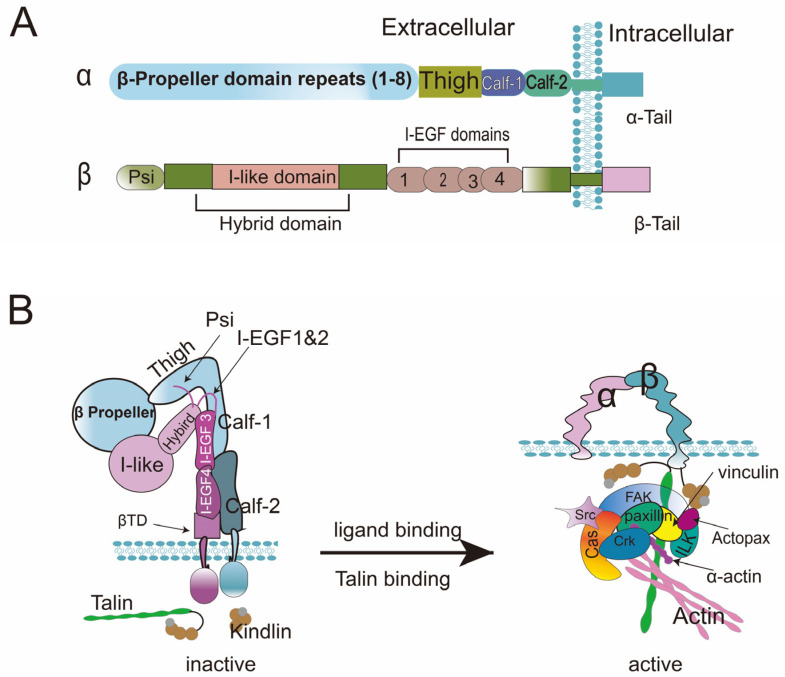
(**A**) **Domain organization and structure of a generic integrin.** α and β Subunits synthesize heterodimeric proteins that adhere to the cell surface. (**B**) **Integrin activation.** In the active state, the recruitment of talin and kindlin by the β subunit’s intracellular domain leads to the extension of the integrin leg and separation of dimeric subunits (at the transmembrane and cytoplasmic domain levels). Upon Talin and kindlin binding to β subunits, intracellular adaptor proteins and enzymes are clustered to form the focal adhesion complex (composed of Src, FAK, Crk, Cas, vinculin, ILK, paxillin, actopax, α-actin, and talin), which in turn strengthens the combination between integrins and ligands. (Cooper et al. [56] have reviewed this in 2019).

Since integrins were discovered in the 1980s, they have taken a central position among receptors involved in cell adhesion and signaling. The integrin family comprises 24 heterodimeric receptors, which mediate cells’ adhesion to a variety of ECM components and to counter-receptors on other cells [55]. The heterodimeric receptors are generally composed of an α subunit and a β subunit, possessing the large extracellular domain and the short intracellular α-tail and β-tail [56]. The extracellular domain of the α subunit mainly contains the N-terminal β-propeller, Thigh domain, and Calf1-2 domains, while the extracellular domain of the β subunit consists of Psi, the hybrid domain, I-like domain, I-EGF1-4 domains, and βTD (Figure 1A). When integrins are not activated, the β propeller in the α subunit combines with the I-like and hybrid domains in the β subunit to form the ligand-binding pocket and the headpiece of the integrin. At this point, the integrin appears in a closed conformation: the ligand binding pocket has a low affinity and faces the plasma membrane and integrin “legs” (α subunits Calf-1 and Calf-2; β subunit I-EGF3, I-EGF4, and the membrane-proximal tail domain βTD) [57] (Figure 1B). Integrin activation requires both ligand and cytoskeletal protein talin or kindlin engagement. Additionally, the association of adaptor proteins (e.g., paxillin, vinculin, α-actinin) and the activation of enzyme molecules (e.g., focal adhesion kinase (FAK), Src) are required to link integrin with actin [58]. Large allosteric changes couple ligand binding to the ligand binding pocket and recruit the cytoskeletal protein to the intracellular portion of the β subunit. The ligated integrins nucleate paxillin, vinculin, α-actinin, FAK, Src kinases, and so forth, which in turn reinforces the connection between cytoskeletal protein and the actomyosin system, increases force transduction across integrins and eventually strengthens integrin–ligand binding. Hence, with the help of adaptor proteins and enzymes, the ligand triggers integrins to combine with the actin cytoskeleton via talin and kindlin, and then activates intracellular signaling. Therefore, it modulates complex cellular behaviors including survival, proliferation, migration, fibrosis, and various cell fate transitions [59,60] (Figure 1B).

#### 3.1.2. Integrins Signaling in Cell Apoptosis, Proliferation, EMT and Fibrotic Process

Mounting evidence has shown that integrins regulate cell proliferation, apoptosis, EMT, and fibrotic processes in eukaryotes. Integrins identify the environment of the cell by interacting with the ECM. When the cellular environment is suitable for survival, integrins binding to the ECM transmit survival signals and enhance cell proliferation. Conversely, integrins disassociated from the ECM trigger apoptosis in a hostile condition. Thus, integrins play roles in maintaining homeostasis [61]. Notably, pro-survival effects mediated by the conjunction of integrins and ECM require intracellular signaling engagement. It is reported that β1 integrin binding to ECM can inhibit the release of cytochrome-c from mitochondria and activate the phosphatidylinositol 3-kinase (PI3K)-AKT signaling, and therefore decreases apoptosis [62]. In addition, integrins are potent regulators of Fas-mediated endothelial apoptosis. Fas (a tumor necrosis factor receptor) binds to the ligand Fas-L and subsequently induces the recruitment of the adaptor protein Fas-associated death domain (FADD) and caspase-8, and eventually activates downstream caspases, leading to irreversible cell death. It is known that integrins regulate Fas-mediated apoptosis at two distinct levels: (a) integrins can directly reduce Fas expression; (b) integrins modulate the expression of c-FLIP, an endogenous antagonist of caspase-8, by binding to the ECM. Consistently, the detachment of integrins and ECM initiates the Fas-Fas-L pathway, which causes cell death or anoikis [63]. Additionally, it has become evident that integrins-mediated cell adhesion to ECM was shown to regulate CyclinD1, CyclinE-cdk2, and Rb protein activities, which favors cell mitosis. The detachment of integrins and ECM arrests cells in the G1 phase and leads to apoptosis [64].

Moreover, it is well-accepted that integrins are essential for the initiation and progression of fibrosis [65,66]. Integrins have been grouped into two main classes according to the predominant mechanism of fibrosis: (a) collagen-binding integrins (e.g., α1β1), which directly sense and control collagen synthesis and deposition; (b) αv integrins (e.g., αvβ3) which regulate ECM deposition by mechano-sensing (ECM stiffness) and modulate the activation of growth factors and additionally influence cellular processes, such as cell proliferation, survival, and differentiation [67,68,69,70]. Collagen-binding integrins play an important role in sensing and maintaining extracellular collagen deposition and structure, which is the central process in fibrosis [71,72,73]. Indeed, a previous study on renal injury showed that TGF-β increased the expression of β1 integrins (α1β1, α5β1 integrins) and ECM components (fibronectin, collagen-I, and collagen-IV) simultaneously, and amplified pathological mesangial ECM accumulation and glomerular fibrosis [74]. In addition, the loss of integrin α3β1 protects mice from bleomycin-induced pulmonary fibrosis in vivo, accompanied by reduced accumulation of myofibroblasts and collagen-1 and attenuated hydroxyproline increase. Mechanistically, integrins α3β1 binds to ECM and strengthens the formation of β-Catenin and Psmad2 complex, which is attributed to accelerated fibrosis [75]. For αv integrins, they have a binding site for arginine glycine aspartic acid (RGD), which allows αv integrins to bind ligands and other adhesion receptors containing RGD sequences, such as fibronectin [76,77]. It is well-established that TGF-β mediated fibrosis in multiple organs. TGF-β was secreted and transported through the endoplasmic reticulum and Golgi apparatus after intracellular ribosome synthesis and was eventually stored extracellularly in an inactive form [78]. To activate TGF-β, αv integrins would combine with the RGD sequence of latency-associated peptide (a TGF-β component). Activated TGF-βphosphorylates Smad then accelerate collagen deposition and fibrosis [79]. In addition, increased expression of αv integrins, especially the αvβ6 and αvβ8, is associated with the worst prognosis of pulmonary fibrosis, liver fibrosis, and renal fibrosis, which is related to higher activation of TGF-β [80,81]. 

Furthermore, recent studies have implicated that integrins were involved in the cellular EMT process [82,83]. It is well-recognized that integrin signaling activation is depending on the formation of adhesion complexes including FAK [84]. The intracellular FAK is activated by integrin αvβ3, and then phosphorylates the downstream PI3K and AKT, which ultimately causes the onset and progression of cellular EMT. Conversely, down-regulation of integrin αv and β3 partially inhibits FAK/PI3K/AKT activation and cell EMT [82,84]. In addition, Wen et al. [83] found that interleukin 32 (IL32), a cytokine with RGD motif, specifically binds to integrin β3 via the RGD motif and activates intracellular downstream p38 MAPK signaling. This signaling significantly amplifies EMT and cell invasion, which is markedly reversed by down-regulating integrin β3 or blocking the p38 MAPK pathway [83].

### 3.2. Cadherins

#### 3.2.1. Structure of Cadherins

**Figure 2:**
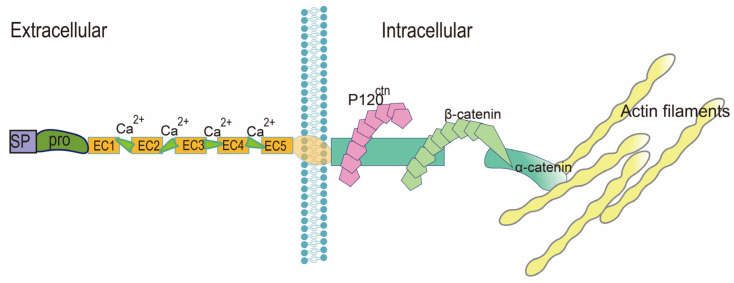
**General structure of cadherins.** Cadherins are single-pass transmembrane proteins including the extracellular domain and cytoplasmic domain. The extracellular domain consists of five homologous repeats (EC1-EC5) (linked by Ca^2+^), SP, and pro. The cytoplasmic domain connects P120^ctn^ and β-catenin, while α-catenin binds to β-catenin to link the cadherin complex and the actin cytoskeleton.

Classical cadherins are transmembrane components of cell adhesive junctions, which mediate cell–cell adhesion through extracellular structures and join the actin cytoskeleton by their cytoplasmic domains. Epithelial cells usually express E-cadherin, whereas mesenchymal cells express N-cadherin, R-cadherin, and cadherin-11. It is well-established that cadherins are important for the establishment of cell polarity during embryonic development [85]. Cadherins are single-pass transmembrane proteins synthesized by a signal peptide (SP) and pro-region (pro), which are removed during protein processing. The extracellular domain contains five homologous repeats (EC1-EC5) linked by calcium ions (Ca^2+^) [86]. The cytoplasmic domain links p120-catenin (P120^ctn^) near the plasma membrane with β-catenin near the C-terminal to form a cadherin complex with the extracellular domain. This cadherin complex then combines with the α-catenin, which is connected with actin filaments [87] (Figure 2). E-cadherin and N-cadherin have similar domains and interactions with catenin [87]. 

#### 3.2.2. Cadherins Signaling in Cell Apoptosis, Proliferation, EMT and Fibrotic Process

E-cadherin is expressed by most normal epithelial tissues and many epithelium-derived cancer cells have lost E-cadherin expression [88]. It is demonstrated that spontaneous apoptosis during gallbladder cancer invasion is closely associated with E-cadherin expression [89]. Moreover, Hsu et al. indicated that the functional expression of E-cadherin in melanoma cells resulted in growth retardation and inhibited cell movement and local invasion in vitro. This effect may partly rely on amplified cell adhesion or modulation of invasion-related cell adhesion molecules (such as β3 integrin) induced by E-cadherin [90]. In addition to the neoplastic epithelium, decreased expression of E-cadherin was also observed in epithelial tissues of some benign lesions [91,92]. In a study on drug-induced kidney injury, Yay et al. [93] observed increased E-cadherin expression and more severe cell apoptosis in mice after Adriamycin (an anthracycline antitumor drug with nephrotoxicity) treatment, It is believed that E-cadherin exerts its growth-suppressive effect directly through tight cell–cell adhesion or indirectly by sequestrating β-catenin [94]. 

Unlike E-cadherin, N-cadherin is upregulated in a variety of cancer cells [95]. Elevated N-cadherin increases cell adhesion and migration ability and exerts an anti-apoptotic effect [96]. Gao et al. treated ovarian cancer cells with pinocembrin, a natural flavonoid with an anti-inflammatory effect, and observed a significant decrease of N-cadherin in the mRNA level in vitro. N-cadherin reduction directly promotes apoptosis and inhibits migration by interfering with cell junctions [97]. Similarly, in a study on ischemic heart failure, N-cadherin overexpression strengthened ADSC (a type of cardiomyocyte) adhesion and migration and enhanced its ability for angiogenesis and cardiomyocyte proliferation. Mechanistically, N-cadherin overexpression significantly increases N-cadherin/β-catenin complex formation and active β-catenin levels in the nucleus. On the other hand, β-catenin knockdown abolishes N-cadherin overexpression-induced matrix metallopeptidase (MMP)-10, MMP-13, and hepatocyte growth factor (HGF) expression and blocks the cellular activities and cardioprotective effects induced by N-cadherin overexpression in ADSC [96]. It is well-known that alterations of cadherins are also a characteristic manifestation of EMT, specifically manifested as E-cadherin decrease and N-cadherin increase [42]. During EMT, epithelial cells lose their typical polygonal and cobblestone forms and acquire spindle-shaped mesenchymal morphology, which favors their migration and invasion. Interestingly, epithelial cells could present plasticity, transient and mixed state, termed as partial EMT phenotype. A partial EMT phenotype has the characteristics of epithelial and mesenchymal phenotype and can conduct collective cell invasion, rather than single-cell migration in mesenchymal cells [98].

It is well-accepted that fibrosis is tightly related to EMT. Epithelial cells can differentiate into fibroblast-like cells, involving wound repair, tissue regeneration, or organ fibrosis with E-cadherin loss, N-cadherin acquisition, increases of fibroblast specific protein-1 (FSP1), α-smooth muscle actin (α-SMA) and collagen [99]. At a cellular level, research on renal tubular fibrosis showed a decrease in E-cadherin and an increase in α-SMA after inhibition of miRNA-34a (a miRNA downregulated by cellular hypoxia). It further found that inhibition of miR-34a activated Jagged1/Notch1 signaling (a pathway regulating cell proliferation, differentiation, and EMT), which mediated the loss of E-cadherin and gain of N-cadherin. Eventually, it enabled cells to acquire a fibrotic phenotype [100]. In addition, TGF-β can also trigger Jagged1/Notch and Hey1/Notch signaling to induce EMT and renal fibrosis [101]. Additionally, the role of cadherins in fibrosis has been well-studied in animal models. Recently, Chang et al. investigated the mouse model of single-walled carbon nanotube(SWCN)-induced pulmonary fibrosis and found an increased occurrence of N-cadherin-positive epithelial-derived fibroblasts at up to 42 days following SWCN-exposure. As fibrosis progresses, the number of proliferative epithelial cells that positively stained TGF-β/pSmad2 or β-Catenin increased, which illustrated that N-cadherin overexpression induced-fibrosis involved the activation of TGF-β/pSmad2 and β-catenin signaling [102].

## 4. Cell Adhesion Molecules in BPH Development

### 4.1. Expression of CAMs in Prostate

As common transmembrane protein receptors, integrins and cadherins are widely expressed in the prostate. Bonkhoff and his colleagues verified the expression of integrin α6β1 and α2β1 proteins in normal, hyperplastic, and neoplastic human prostate tissue by the avidin-biotin complex method two decades ago [103]. Moreover, the basal cells of the normal prostate gland express several integrin alpha units including α 2,3,4,5,6,7 and v [104,105]. Some beta subunits such as β1 and β4 are observed in normal prostate as well. These integrin units are polarized at the base of the cells where they co-distribute with the surrounding extracellular matrix [105]. In addition, analysis of primary human prostate cancer cells isolated from 16 surgical specimens showed that PCa cells expressed integrin αvβ3 while normal prostate epithelial cells did not [106]. Additionally, the subunit β6 is upregulated in adenocarcinoma and metastases but absent in normal cells [107]. With regard to cadherins, it is demonstrated that E-cadherin expression was reduced in BPH compared with normal prostate tissues [91]. Additionally, Drivalos et al. used immunohistochemistry to analyze the expression of N-cadherin in the prostate and found increased expression of N-cadherin in PCa tissue compared with normal prostate tissue [104].

### 4.2. CAMs and Cell Proliferation/Apoptosis Ratio

**Figure 3:**
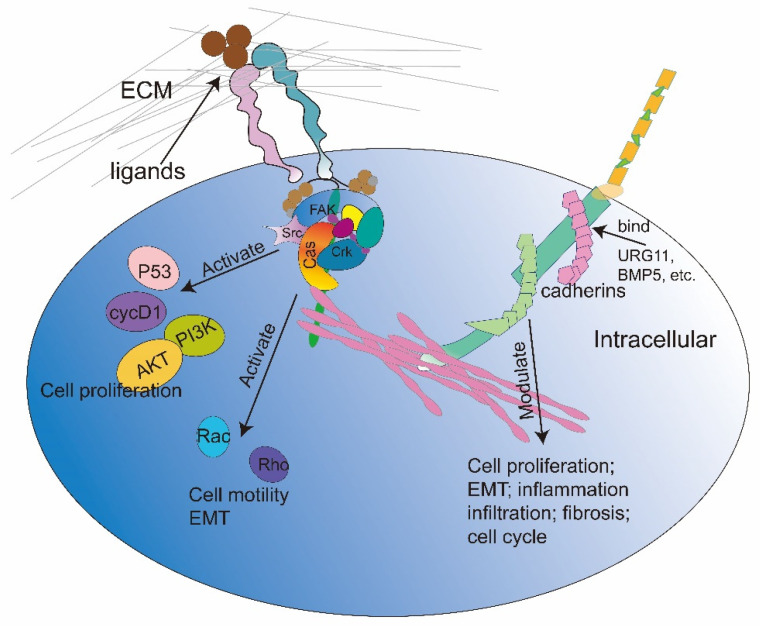
**CAMs on prostate cells:** ECM or ligands combine with integrin to recruit talin, paxillin, FAK, Src, and other proteins to form a complex. The integrin complex further regulates cell proliferation and apoptosis by regulating downstream PI3K-AKT signal, cycD1 activation, and P53 expression. In addition, the complex could directly affect the activity of Rho and Rac to regulate cell migration and EMT. Some molecules such as URG11 and BMP5 bind to E-cadherin and/or N-cadherin in prostate cells. After binding those molecules, cadherins can directly regulate cell proliferation, EMT, inflammation, fibrosis, and the cell cycle.

The proliferative and apoptotic imbalance of cells is the core pathophysiology of prostatic hyperplasia. As aforementioned, the regulatory roles of CAMs (such as integrins and cadherins) in proliferation and apoptosis have been identified in a variety of diseases. More precisely, the pro-survival signals delivered by the link between the integrins and the ECM could favor cell survival and proliferation. The up-regulation of N-cadherin increases cell adhesion and migration, along with proliferation ability, while the elevated expression of E-cadherin induces cell apoptosis. It was reported that integrin α2β1 and collagen-I interaction initiated the activation of MAPK kinase 7 (MAPKK7), resulting in cell proliferation and EMT in vitro, whereas in the presence of the integrin inhibitor BTT-3033, an inhibitor that interferes with the interaction between integrins and ECM, MAPKK7 phosphorylation was inhibited, leading to cell apoptosis and cell cycle arrest at the G1 phase [108]. In another study, BTT-3033 exhibited a survival inhibitory effect on prostate stromal cells WPMY-1 at high concentrations (10 µM) [109]. Similarly, a decreased integrins expression and an increased apoptotic ratio were observed after treatment with doxazosin in the BPH-1 cell line, suggesting the therapeutic effects of doxazosin on BPH partly rely on the loss of integrins [110]. Furthermore, as shown in Table 1, in addition to integrin α2β1, studies have shown that there are several integrins that promote cell growth, invasion, and migration in prostate cells (benign or malignant) (Table 1). Mechanistically, after the connection between integrins and ECM, talin and kindlin are recruited and bound to the tail of the β subunit. Upon the first binding, the integrin intracellular domain further binds and activates FAK and Src with the help of talin and paxillin. Activated FAK and Src recruit the Cas-Crk complex, which promotes Rac and Rho GTPases activity and thus regulates cell migration. In addition, FAK could affect cell survival and proliferation by affecting PI3K-AKT activation, cycD1 expression, and kinase-independent effects on p53 activity [56,111] (Figure 3). It has also been reported that E-cadherin expression was lowered in BPH prostate, which was associated with prostate cell proliferation directly or indirectly through inflammation and collagen accumulation. To date, accumulating evidence has revealed the relationship between E-cadherin and prostate cell survival. In an in vivo study, *CDH1* (a gene encoding E-cadherin) knockout mice (CDH1+/−) exhibited prostatic stromal hyperplasia, inflammation infiltration, ECM deposition, and interstitial fibrosis [112]. Consistently, Pascal et al. [113] observed that conditional deletion of E-cadherin in luminal prostate epithelial cells resulted in prostatic epithelial and stromal hyperplasia, which was related to the infiltration of CD19-positive B cells, CD68-positive macrophages, and CD3-positive T cells. As for N-cadherin, it is encoded by the *CDH2* gene. Quan et al. uncovered that N-cadherin up-regulation exerted a pro-survival role in the prostate, whereas opposite effects of cell proliferation, invasion, and migration were observed when *CDH2* silenced [114]. Notably, there are couples of genes upstream of cadherins, such as upregulated gene 11 (*URG11*) [115] and *BMP5* [40], which is involved in the biological processes of multifarious diseases. Zhang et al. demonstrated that silencing *URG11*, a gene upregulated by hepatitis B virus X protein, suppressed the expression of cyclin D1, N-cadherin, and vimentin whereas upregulated the levels of E-cadherin and p27 in prostate epithelial cells BPH-1. Therefore, it inhibited cell proliferation and induced cell cycle arrest. Moreover, treatment with TGF-β in BPH-1 cells obviously increased the level of URG11 mRNA and presented a pro-proliferative effect [115]. In our earlier study, we used siRNA to knock down *BMP5* in WPMY-1 and BPH-1 and observed increased E-cadherin expression, decreased N-cadherin expression, cell cycle arrest, and cell proliferation inhibition [40] (Figure 3).

### 4.3. CAMs and Prostate Smooth Muscle Contraction, EMT, and Fibrosis

Prostatic smooth muscle contraction is essential for the development and management of BPH-associated LUTs. Integrins connect the cytoskeleton to the membrane and then connect the cell to the ECM, which directly induces force generation for smooth muscle [116]. In an organ bath study, integrin inhibitor BTT-3033 attenuated prostate smooth muscle contractions induced by electric field stimulation and various doses of thromboxane A2 analogue U46619 by 46% and 39%, respectively. Thus, it is suggested that inhibition of integrins may lessen prostate smooth muscle contraction and then relieve LUTs [116]. Regarding cell adhesion, the extracellular domain of E-cadherin is tightly connected to cells or ECM. Therefore, it guarantees the integrity of the prostatic luminal epithelial barrier. Indeed, it has been demonstrated in vivo and in vitro that E-cadherin knockdown increased monolayer permeability and disrupted the formation of cellular tight junctions, which produced chronic leakage of secreted prostatic proteins into the basement membrane and stromal compartment and potentially contributed to the occurrence and progression of BPH [91]. As the hallmark proteins of EMT, cadherins also regulate the cellular EMT process and prostate fibrosis. Pascal et al. showed that mice with E-cadherin deletion developed macrophage inflammation, increased stromal proliferation, and bladder overactivity, indicating that E-cadherin deficiency may promote inflammation infiltration and fibrosis in the prostate [112] (Figure 3). However, no evidence has shown that integrins can regulate EMT in prostate hyperplasia, although they can promote EMT in many neoplastic diseases, of which further investigation would be intriguing [117].

**Table 1:** Effects of CAMs on the prostate.

CAMs	Effects	References
Integrin α2β1	Amplifying proliferation; promoting EMT; strengthening prostate smooth muscle contraction	[108,109,116]
Integrin α3β1	Heightening cell adhesion and cell proliferation	[118]
Integrin α4	Heightening cell adhesion and cell migration	[119]
Integrin α5β1	Amplifying migration and invasion; promoting survival	[120]
Integrin α6β1	Promoting cell survival	[121,122]
Integrin αvβ3	Strengthening cell migration and proliferation	[123,124]
Integrin αvβ6	Amplifying migration and invasion; promoting survival	[125,126]
E-cadherin	Reducing fibrosis and inflammation infiltration; anti-proliferation; attenuating EMT	[113,127]
N-cadherin	Promoting cell survival; enhancing EMT	[114,127]

## 5. CAMs as Potential Therapeutic Targets for BPH

Currently, oral therapies for BPH include alpha-receptor blockers, phosphodiesterase 5 inhibitors (PDEIs), and 5-alpha-reductase inhibitors (5ARIs) [128]. Alpha-receptor blockers (e.g., doxazosin, tamsulosin) inhibit prostate smooth muscle contraction mediated by norepinephrine [129]. Different from alpha-receptor blockers, PDEIs could reduce the breakdown of cGMP to decline smooth muscle tone in the prostate [130]. Moreover, 5ARIs (i.e., finasteride, dutasteride) attenuate the conversion of testosterone to the more active dihydrotestosterone (DHT) and thus trigger apoptosis of the prostatic epithelium, leading to atrophy of the prostatic gland [131]. Although all these agents are effective and acceptable, they are not always able to prevent the progression of BPH. Interestingly, CAMs have recently been recognized as promising molecular targets for the treatment of many diseases. 

Integrin inhibitors, including BTT-3033, abituzumab, and mAb 33B6, have been studied in BPH and PCa treatment. It was reported that BTT-3033 could lessen prostate smooth muscle contraction and relieve BPH by the dissociation of ECM and integrins [116]. Similarly, as a monoclonal antibody (mAb) targeting integrin αv submit, abituzumab could inhibit the activation of many integrin signaling pathways, such as FAK and AKT, which have been demonstrated to promote cell growth and tumor growth [132]. Additionally, this antagonist could inhibit cell–cell interactions, cell-to-ECM interactions, cellular invasion, cell migration, and cell signaling. Based on abituzumab-induced recognition inhibition between integrin αv extracellular domain and ligands, it exerted an antitumor activity [133]. In another study, the 33B6 mAb was developed as an inhibitor of activated conformation of β1 integrin. Lee et al. [134] treated PCa cells with the 33B6 mAb and observed decreased adhesion of cell-ECM and increased apoptosis by 3-fold, suggesting the therapeutic effect of the 33B6 mAb in PCa.

In addition, targeting E-cadherin and N-cadherin has been demonstrated as an emerging therapeutic strategy. Bajrami and his colleagues [135] have reported that ROS1 inhibitors (such as crizotinib or foretinib) could induce mitotic abnormalities and multinucleation in E-cadherin-deficient cells, which could be attributed to the defect in cytokinesis and aberrant p120^ctn^ phosphorylation and localization. It finally resulted in increased apoptosis and exerted antitumor effects. Moreover, Rho effector kinases such as myotonic dystrophy kinase-related cdc42-binding kinases α (MRCKα) also possess synthetic lethal in E-cadherin defective cells, which showed potential to treat E-cadherin deficient diseases, such as BPH [136]. With regard to N-cadherin, its antagonists mAbs were capable of inhibiting cell–cell adhesion [137]. Previous studies have implicated that these kinds of mAbs directly antagonized the N-cadherin EC domain and therefore inhibited cancer cell proliferation and invasion both in vitro and in vivo [138,139]. Meanwhile, another N-cadherin antagonist, ADH-1, has been shown to block N-cadherin-mediated adhesion, migration, and proliferation in multiple cell types in vitro [140,141,142]. ADH1-induced adhesion loss could enhance cell apoptosis, which was associated with the downregulation of PI3K/AKT/Bad pathway signaling [139]. Moreover, in an in vivo study, a Chinese herbal extract antoridan could down-regulate N-cadherin, up-regulate E-cadherin, and effectively inhibit EMT, collagen deposition, and prostate fibrosis. It finally could alleviate most of the BPH pathological symptoms [143]. 

However, only a small number of drugs have been studied in BPH and showed therapeutic effects of alleviating BPH/LUTS. In addition, the side effects need to be fully considered and addressed. Therefore, a large number of scientific studies are still necessary to further evaluate the efficacy and safety of those agents.

## 6. Conclusions

In aggregate, CAMs are tightly related to the occurrence and development of BPH. Alterations in CAMs mediate cell-ECM and cell–cell tight junction, activate TGF-β/Smad signaling, promote inflammatory infiltration, and prevent cell cycle arrest. These signals then further alter the cell proliferation/apoptosis ratio, promote EMT and prostate fibrosis, and enhance the contraction of smooth muscle in the prostate, finally accelerating the progression of BPH/LUTS. On the other hand, blocking CAMs is perhaps an emerging therapeutic target for BPH. The development of new drugs targeting specific CAMs will become increasingly important in the coming years.

## Data Availability

Not applicable.
